# The buccal minor salivary glands as starting point for a metastasizing adenocarcinoma – report of a case

**DOI:** 10.1186/1746-160X-4-16

**Published:** 2008-07-30

**Authors:** Tobias Ettl, Johannes Kleinheinz, Ravi Mehrotra, Stephan Schwarz, Torsten Eugen Reichert, Oliver Driemel

**Affiliations:** 1 Department of Oral and Maxillofacial Surgery, Regensburg University, Germany; 2 Department of Oral and Maxillofacial Surgery, Muenster University, Germany; 3 Department of Pathology, Moti Lal Nehru Medical College, Allahabad University, India; 4 Department of Pathology, Erlangen University, Germany; 5 Department of Cranio-Maxillofacial Surgery, University Hospital Muenster, Waldeyerstr. 30, D-48149, Muenster, Germany

## Abstract

**Background:**

With the 2005 WHO classification of salivary gland tumours and its increasingly recognized diagnostic entities, the frequency of adenocarcinoma (NOS) has decreased significantly.

**Case presentation:**

This paper describes a fast growing adenocarcinoma (NOS), originating from the minor salivary glands of the left buccal mucosa with a rapid onset of multiple local and distant metastases, especially in the lung. A lung primary was unlikely as the tumour was characterized by positivity for cytokeratin 20 and negativity for the thyroid transcription factor-1 protein (TTF-1) in immunohistochemistry.

**Conclusion:**

A rare case of an adenocarcinoma (NOS) of the minor salivary glands with a rapid development and an unfavourable clinical course is reported. It shows that additional immunohistochemical analysis can decisively contribute to determine the site of the primary tumour in cases with unknown primary.

## Background

Epithelial tumours arising in the intra-oral minor salivary glands account for 9–23% of all salivary gland tumours [1,2] and of these, carcinomas are responsible for about 40–54% [3-5]. Adenocarcinoma not otherwise specified (NOS) is a malignant neoplasm of the salivary glands with ductal, glandular or secretory differentiation that cannot be attributed to any other currently recognized type of salivary gland carcinoma [6,7]. With the 2005 WHO classification of salivary gland tumours and its increasingly recognized diagnostic entities, frequency of adenocarcinoma (NOS) has decreased significantly [7]. This article describes a fast growing adenocarcinoma (NOS), originating in the left buccal mucosa with a rapid onset of multiple local and distant metastases. Immunohistochemistry was found to be useful in confirming a salivary gland origin.

## Case presentation

A 68-year old female patient with a painless swelling of the left buccal mucosa was referred to our department. An initial incisional biopsy of the lesion was inconclusive and magnetic resonance imaging (MRI) of the head and neck diagnosed a benign appearing connective tissue tumour, arising without local invasion.

Detailed medical history pointed to a more than three months consisting, rapidly enlarging mass in the patient's left buccal mucosa, which provoked pain while using her dentures. The patient further complained of lack of appetite, sleeping disturbance and weight loss of 11 kilograms (15% of body weight) over the last five months. Tobacco and alcohol abuse was excluded.

**Figure 1:**
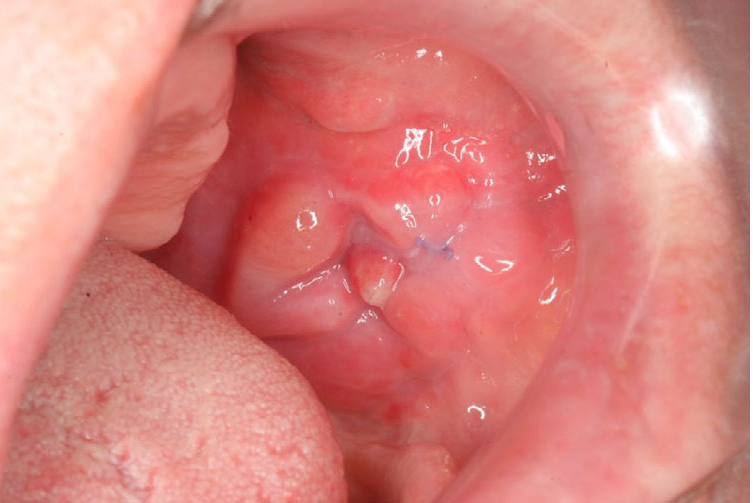
**Intraoral finding after initial biopsy: Exophytic and endophytic growing tumour of the left buccal mucosa (3 × 2 × 1.5 cm^3^) with indiscernible borders**.

Intraoral examination revealed an asymptomatic, solid, firm, exophytic and endophytic growing tumour of the left buccal mucosa (Fig 1). The tumour was fixed to adjacent structures and extended caudal to the mandible. Examination of the patient did not reveal facial paralysis, paraesthesia and palpable regional lymphadenopathy. Haematologic parameters were all within normal range. For further elucidation, a deeper biopsy was performed. During surgery, the tumour could hardly be separated from the surrounding connective soft tissue and adjacent alveolar bone. The retromolar alveolar crest appeared disintegrated and was suspicious of bone invasion, so a specimen of the alveolar bone was taken as well.

**Figure 2:**
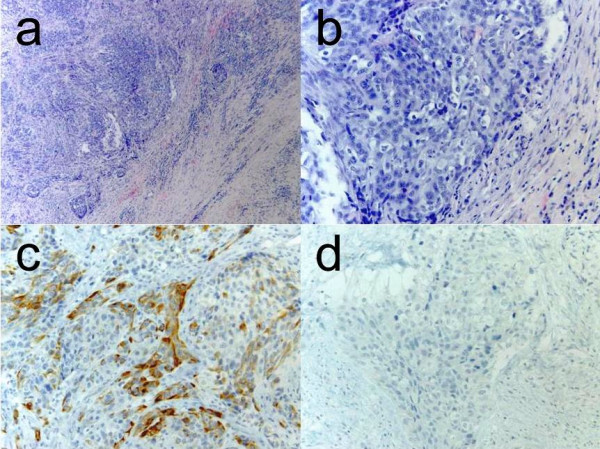
**Histopathology**. a: Tumour with solid and invasive growth pattern surrounded by desmoplastic connective tissue (H&E, 40×). b: in detail: Hyperchromatic, pleomorphic nuclei with necrosis and numerous mitoses (H&E, 200×). c: Positive immunohistochemical staining for Cytokeratin 7 (CK7, 200×). d: Negativity for the thyroid transcription factor 1 (TTF-1, 200×).

Histopathological analysis of the specimen, supported by immunohistochemistry (CK7 and CK20 positive; CK5/6, Aktin and HER 2 negative) allowed the diagnosis of a poorly differentiated adenocarcinoma (NOS) of the minor salivary glands (Fig 2a–c).

**Figure 3:**
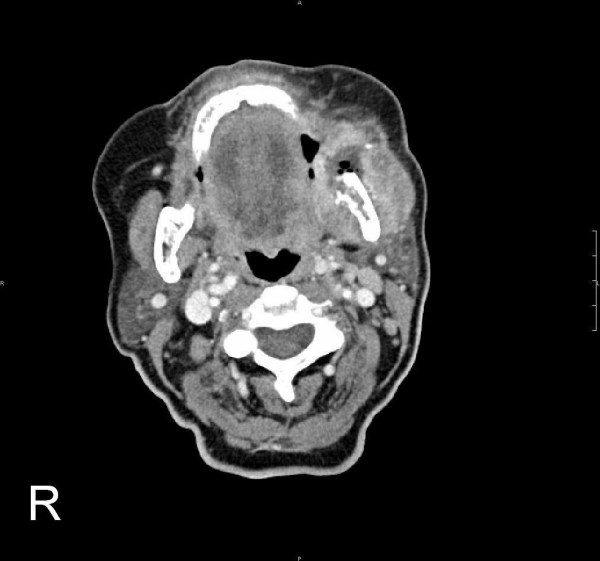
Computerized tomography (CT) with contrast medium (CM): Axial image of the head and neck: Tumour (4 × 5 cm^2^) of the left buccal soft tissues with central necrotic and partly calcified components and resorption of the left mandible.

**Figure 4:**
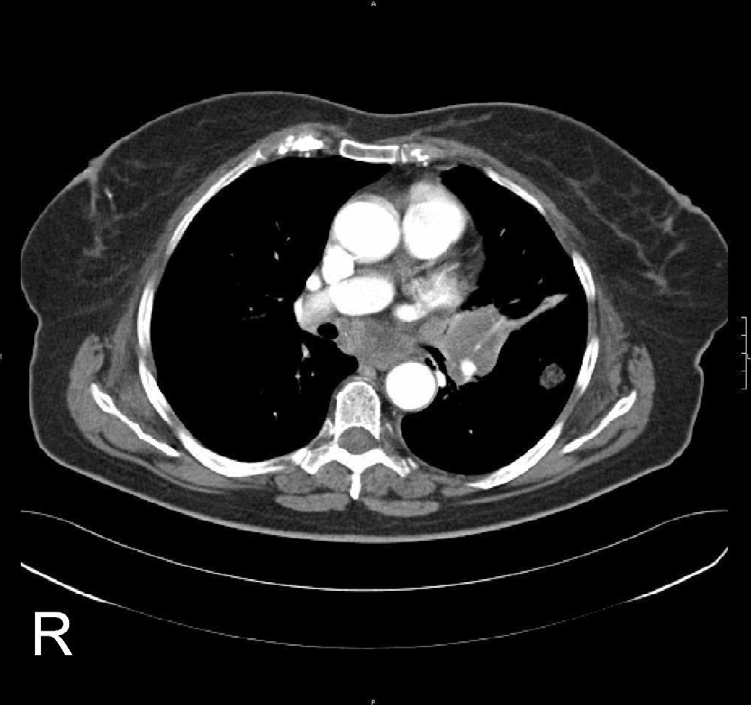
Chest: Left hilar mass (4.5 × 4.4 cm^2^). Local infiltration into mediastinum; additional mass on the left side.

**Figure 5:**
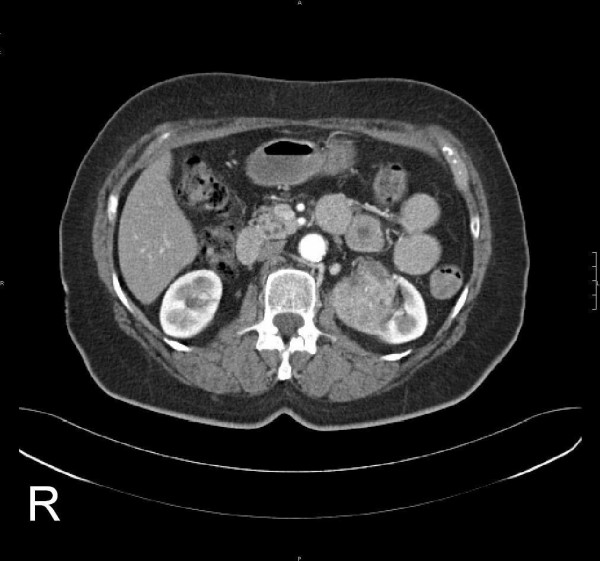
Abdomen: Left renal tumour (4.6 × 4.1 cm^2^).

**Figure 6:**
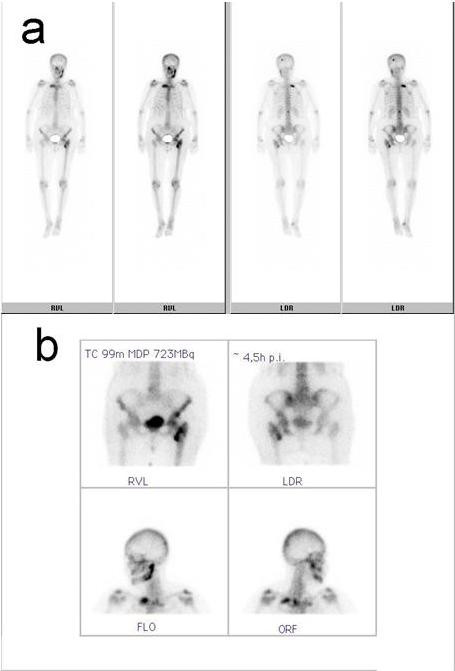
Bone scan. a: Total body, b: Head-neck-SPECT-image: For metastasis suggestive accumulation of 99mTc in the calvarium, the left mandible, the second rib, the second lumbar vertebral body and the left hip.

Positron-emission tomography with 'low dose CT' (PET-CT), computerised tomography (CT head and neck, chest, pelvis and abdomen) and bone scan showed the tumour in the left buccal area and an additional circular mass in the hilum of the left lung, a tumour of the left kidney, as well as multiple pulmonary, cervical lymph nodes and osseous (skull, spine, rib, pelvis) masses (Fig 3, 4, 5, 6). Bronchoscopic biopsy of the hilum mass also identified a poorly differentiated adenocarcinoma (NOS). Since the immunohistochemical analysis was negative for the thyroid transcription factor-1 protein (TTF-1) (Fig 2d) and was positive for cytokeratin 20, a primary adenocarcinoma of the lung was unlikely and the tumour was finally attributed to the minor salivary glands as site of origin.

Due to the extent of the disease, palliative chemotherapy was initiated.

## Discussion

Data concerning the relative frequency of adenocarcinoma (NOS) vary from 1.2% to 17.8% of all salivary gland carcinomas [6,8], since in previous classifications tumours, which are currently established as more specific histologies like salivary duct carcinoma, epithelial-myoepithelial carcinoma or polymorphous low-grade adenocarcinoma, were often categorized as adenocarcinoma (NOS) [6,8]. About 40% of adenocarcinomas (NOS) are located in the minor salivary glands [7], with a relative frequency of 4.3%–10.3% of all minor gland carcinomas [3-5]. The palate is the most commonly involved site (39%–75%), followed by the lips and the buccal mucosa, as described in the case report [3,4]. In most cases, the lesion presents as a firm, solid and painless mass, which may be characterized by ulceration and fixation to the surrounding soft tissues. Mechanical irritation like friction from the patient's denture may evoke tenderness.

In general there are various differential diagnoses for a buccal swelling comprising both benign and malignant neoplasia. Tumours may originate from the squamous epithelium (papilloma, squamous cell carcinoma), the soft tissue (fibromatosis, nodular fasciitis, malignant fibrous histiocytoma, fibrosarcoma, leiomyoma, leiomyosarcoma, lipoma, liposarcoma, neurofibroma, schwannoma, malignant peripheral nerve sheath tumour, hemangioma, angiosarcoma) and from salivary glands (pleomorphic adenoma, adenoid cystic carcinoma etc.) [9,10]. In view of the fact that the majority of Non-Hodgkin's lymphomas affecting the oral cavity present as a submucosal mass, this differential diagnosis should also be taken into account, although the hard palate and the gingiva are the most common intraoral sites of occurrence [10]. Oral metastatic lesions can also be the initial appearance of undiagnosed primary malignancies. Because of the rapid growth of the tumour, its firm appearance and spread to adjacent structures, its intraoperatively obvious bony invasion and considering the patient's history (lack of appetite and weight loss), a malignancy was the most likely diagnosis in the present case.

Microscopically, adenocarcinoma (NOS) is characterized by a variable spectrum of different architectural patterns, which may include glandular, papillary, cystic, cribriform or solid structures [6]. Tumours with considerable heterogeneity of growth patterns, which cannot clearly be attributed to well known entities of adenocarcinoma should best be classified as adenocarcinomas (NOS). According to the most recent WHO classification, tumours showing a high morphologic heterogeneity, a low mitotic rate and slight nuclear atypia can better be assessed as polymorphous low-grade adenocarcinoma. Hence, the majority of adenocarcinomas will be of high malignancy grade, as in this case, characterized by hyperchromatic and pleomorphic nuclei, necrosis and high mitotic rate [7]. Adenocarcinomas with overt presence of ductal structures should better be classified as salivary duct carcinoma (SDC) than as adenocarcinoma NOS, but the distinction might be arbitrary. Immunohistochemistry may help, as more than 90% of SDCs are specifically positive for androgen receptors (AR) and because most of these carcinomas show positive staining for HER-2/neu (c-erbB-2) [11].

Cytokeratins (CK) are distinctive intermediate filaments, which are confined to epithelia and indicate the tissue of origin in malignant transformation and metastasis [12]. They may also be useful in the determination of the primary site. While CK 5/6 is common in squamous epithelia, the expression of CK 7 and CK 20 is distinctive in glandular epithelia. This may include tumours like colorectal, pancreatic or bronchioloalveolar adenocarcinoma as well as adenocarcinomas of the salivary glands [13]. Since the patient in this case report presented with an additional adenocarcinoma of the lung, the primary site of the carcinoma had to be elucidated, especially oral metastasis by a lung primary had to be excluded. The thyroid transcription factor 1 (TTF-1) is a specific marker of the thyroid gland and the epithelia of the lung, regulating the expression of surfactant in the latter organ [14,15]. Evidence of antibodies to TTF-1 may identify the lung as the primary site of origin in adenocarcinoma with unknown primary. In the reported case TTF-1 turned out to be negative. Together with the positivity for CK20 which is usually negative in primary adenocarcinomas of the lung, a salivary gland origin was most likely. Immunohistochemistry might also aid in the differential diagnosis of salivary gland carcinoma types. In the present case the tumour cells were negative for CK5/6, a marker of basal cells, myoepithelial cells and squamous epithelium excluding a variety of carcinoma types: mucoepidermoid carcinoma, squamous cell carcinoma and myoepithelial carcinoma.

The overall prognosis of adenocarcinoma (NOS) depends on clinical stage and malignancy grade. For stage I a 10-year survival rate of 75% has been reported by Spiro et al [16], dropping to 36% for stage II, irrespective of grade. According to the same study 15-year survival rates for low-, intermediate- and high-grade adenocarcinoma are 54%, 31% and 3% respectively [16]. However, this study most likely includes tumours, which are today, further subclassified. Tumour site has also been mentioned to govern the prognosis. Carcinomas of the oral cavity are reported to have a more favourable outcome (76% at 10 years) than those of the parotid (26% at 10 years) or the submandibular glands [17]. In a study of 54 patients with adenocarcinoma (NOS) of the major and minor salivary glands, cervical lymph node metastases were recorded in 23% of the patients and distant metastases developed in 37% of these patients [17].

## Conclusion

Although incidence of the adenocarcinoma (NOS) is decreasing with the establishment of new neoplastic entities of the salivary glands, this carcinoma still occurs and should be taken into account in case of intraoral mucosal tumours with indiscernible borders. High-grade malignancies arising in the minor glands may show a rapid growth and early metastases to lymph nodes and distant organs. Additional immunohistochemical analysis can decisively contribute to determine the site of the primary tumour.

## Competing interests

The authors declare that they have no competing interests.

## Authors' contributions

TE drafted the manuscript. JK helped to the critical review of the article. RM helped to the critical review of the article. SS performed the histopathological investigations. TER helped to the critical review of the manuscript. OD performed the surgical procedure, helped to draft the manuscript, helped to the critical review of the manuscript.

## Consent section

Written informed consent was obtained from the patient for publication of this case report and accompanying images. A copy of the written consent is available for review by the Editor-In-Chief of this journal.
